# Measurement and evaluation of pesticide residue risk in vegetables: evidence from China

**DOI:** 10.3389/fpubh.2025.1563272

**Published:** 2025-05-23

**Authors:** Taiping Li, Xiaoshan Jiang, Menghui Qiu

**Affiliations:** College of Economics and Management, Nanjing Agricultural University, Nanjing, China

**Keywords:** pesticide residues, risk assessment, food safety, supervision and inspection data, vegetables

## Abstract

**Introduction:**

With the modernization of agricultural production and the widespread use of chemical pesticides, pesticide residue risk has emerged as a significant public health concern globally. Pesticide residues in vegetables represent a potential hazard to consumer health. A scientific evaluation of their risk status not only enhances information transparency but also provides a precise foundation for food safety regulation.

**Methods:**

This study devised a vegetable pesticide residue risk index by integrating the probability of residue detection and the extent of associated harm, utilizing 294,703 monitored sampling data across 30 provinces from 2021 to 2023 to methodically assess and quantify the risk of vegetable pesticide residues in China.

**Results:**

Out of 52 vegetable types analyzed, 33 had pesticide residue levels that surpassed the permissible limits, with leeks and celery registering the highest risk indices. Regarding regional variations, Jiangsu, Jilin, Hubei, Hainan, and Heilongjiang were identified with the highest risk levels, while Ningxia, Yunnan, Shaanxi, Gansu, and Tianjin presented the lowest. The dispersion of vegetable pesticide residues has been progressively extending from the northeast towards the southwest. The predominant pesticide residues were found in three types—Clothianidin, Procymidone, and Chlorpyrifos, which constituted 43.6% of the overall exceedances.

**Discussion:**

These findings provide a scientific basis for risk-based regulation, supporting region-specific inspection and targeted pesticide control. The study advocates tailored regulatory measures that consider both regional risk profiles and pesticide characteristics to reduce residue risks. Enhanced data transparency further empowers consumers to make informed choices, fostering a bottom-up compliance mechanism.

## 1 Introduction

Food safety risk is a ubiquitous challenge faced by nations globally (1, 2) with pesticide residue constituting an inescapable issue within this domain (3). As staple components of the daily diet, the risk associated with pesticide residues in vegetables has escalated into a widespread public health concern (4). China, as the leading vegetable producer globally, accounts for over half of the worldwide output, boasting an average annual per capita consumption of vegetables at 515 kg—more than triple the global average (5). Historically, the excessive use of pesticides in China’s agricultural sector has led to vegetables becoming notably problematic due to their brief growth cycles and limited capacity for pesticide degradation (6). Concurrent with rapid economic advancement and rising living standards, there is a growing consumer demand for premium-quality vegetables, thereby accentuating the prominence of pesticide residue risks (7, 8). According to the “2023 China Modern Diet Development Index, “pesticide residues are the foremost among the “top 10 food safety concerns for the Chinese populace.” Data from the State Market Supervision and Administration Bureau reveal that between 2020 and 2023, the proportion of non-compliant vegetable batches among edible agricultural products rose from 42.7 to 54.49%, with over 70% of these violations linked to pesticide residues (9). Pesticide residues impart considerable negative externalities, posing significant health risks through accumulation in the food chain and bioaccumulation effects. Due to the credence attributes of agricultural products, it is challenging for consumers to ascertain pesticide residue levels before and after purchase (10, 11).

Food safety risks increasingly accumulate, intersect, and converge, potentially breaching critical thresholds that could precipitate food safety crises and imperil human health (12). Consequently, it is imperative to mitigate these risks through proactive management practices to forestall the escalation of food safety risks into more severe problems (13). Nonetheless, the rising complexity and diversity of food-related issues (14), mean that traditional risk-based food safety methods—predominantly reliant on regulatory inspections and sampling—no longer suffice to guarantee consumer safety (15). Internationally, food safety management has evolved toward a more sophisticated, risk-based framework for food safety control (16). It is broadly acknowledged that the cornerstone of efficaciously preventing and controlling food safety risks lies in comprehending the fundamental dynamics of risk evolution through meticulous risk assessment and analysis. Concurrently, given the constraints of China’s supervisory resources (1), the inadequacies of regulatory measures, and the subjective and delayed nature of risk monitoring (11), it becomes crucial to initially prioritize the monitoring of pollutant supervision in foodstuffs (17). Thus, food safety risk analysis has emerged as a potent method to restructure the food safety supervision system and address food safety challenges effectively (18). The resolution of vegetable quality and safety issues hinges on precisely understanding the current state and characteristics of pesticide residue risks and on the scientific distribution of regulatory resources (19). Accurate identification of pesticide residue risks in distinct vegetable categories forms the foundation for optimizing the allocation of regulatory resources. Furthermore, quantitative identification and evaluation of food safety risks enable a more focused determination of the principal causes, vulnerable points, and critical control junctures of food safety issues. This approach not only enhances market transparency and furnishes consumers with dependable safety information, thereby steering market selection and promoting equitable competition, but it also offers a scientific basis for the government to devise and enforce precise regulatory policies to bolster food safety. Nevertheless, systematically identifying and evaluating the risk of pesticide residues remains a significant challenge that demands resolution.

While numerous hazard identification tools have emerged recently, studies indicate that varying risk assessment methodologies yield disparate outcomes (20). In the realm of pesticide residue risk assessment for consumable agricultural products, existing research predominantly concentrates on three dimensions: the likelihood of pesticide residues exceeding safety thresholds, the severity of potential harm, and the assessment of dietary risks. First, research has quantified the risk levels of pesticide residues in consumable agricultural products by analyzing the likelihood of residue occurrence, utilizing indicators like the detection rates of highly toxic pesticides or the exceedance rates of low-toxicity residues (9). Notably, Zhu et al. (21) conducted a comprehensive review of literature on pesticide residues in Chinese vegetables from 2012 to 2016, elucidating the status and trends within this domain. Zhou et al. (22) assessed the pesticide residue scenario in China’s market by examining two pivotal metrics—the number of non-compliant batches and the non-compliance rate—drawing on data from the National Market Supervision and Administration spanning 2011 to 2020. In a parallel study, Liang et al. (23) evaluated pesticide residues that exceeded standards in U.S. agricultural products, employing the failure rate index based on data from the U.S. Food and Drug Administration (FDA) covering 2009–2017. Furthermore, Li et al. (24) investigated vegetable sampling data in China from 2014 to 2017, revealing that at least one pesticide residue was detected in 82.67% of the samples. Secondly, several studies have utilized the Toxicity Exposure Ratio (TER) or Risk Quotient (RQ) to quantify pesticide residue risks based on hazard levels. The TER, a metric of the toxicity threshold relative to actual exposure, indicates that a lower TER corresponds to higher risk, as demonstrated by Vaíková et al. (25) in their analysis of residual pesticide risks in soil. Conversely, the Risk Quotient (RQ) calculates potential health risks by comparing the exposed population’s dose to the reference dose, with higher RQ values signaling greater risks. Mu et al. (26) used RQs to assess ecological risks posed by mixed pesticides to soil biota, while Zuo et al. (27) evaluated pesticide residue risks in vegetable soils by comparing measured values to maximum residue limits. Additionally, Mac et al. (28) developed a food quality index based on the aggregate of ratios between pesticide concentrations and corresponding maximum residue limits, providing a measure of overall food quality. Other research includes analysis of two reports by the Brazilian government—the Program for Analysis of Residues of Pesticides in Food (PARA) and The National Program for Control of Waste and Contaminants (PNCRC). These studies compared the theoretical maximum daily intake (TMDI), calculated by multiplying the maximum residue limit (MRL) in food by consumption (F), with the Pesticide Residue Sample Index (PRSI), derived from pesticide residues measured in crops across various regions of Brazil, also multiplied by consumption (F). This approach facilitated the identification of pesticide residue risks in Brazilian crops (29). Finally, research has been conducted to evaluate the potential health risks associated with pesticide residues through dietary exposure assessments. The primary methods of evaluation include deterministic and probabilistic distribution approaches. The deterministic method quantifies risk as a function of toxicity and exposure, defined as dietary exposure risk = (residual concentration × dietary intake) /health guidance value (30). Tong et al. (31) applied this deterministic approach to assess the dietary exposure of 68 pesticides in vegetables consumed in Shanghai. Similarly, Lin et al. (32) and Abdo et al. (33) estimated pesticide residue levels in vegetables from Zhejiang and Jordan, respectively, and determined their potential health impacts by integrating data on consumer eating patterns with regional pesticide residue detection results. The probabilistic assessment method, on the other hand, employs the construction of probability distributions for food consumption and pesticide residue concentrations, utilizing Monte Carlo simulation to gage food safety risks (34–36). In summary, while existing studies have contributed significantly to understanding pesticide residue risks, they exhibit notable limitations. Firstly, risk is a complex function of the probability of occurrence and the degree of harm, entailing uncertainties and potentially severe consequences (37). Research often employs singular evaluation indices based on either the likelihood of occurrence or the severity of potential harm, which may not adequately reflect the true risk profile and real-world conditions of pesticide residues. Secondly, risk assessment models, especially those used in dietary risk evaluations, tend to rely on limited sample data or experimental findings from specific regions and populations. Such data may not adequately represent variations in consumption patterns, quantities, and physiological differences across diverse populations, leading to inherent flaws in terms of representativeness and generalizability. This issue is particularly acute in countries like China, where vegetable consumption markedly differs across regions. Moreover, because of the cross-sectional nature of most studies or experimental approaches, dietary risk assessments typically focus on current exposure levels, thereby neglecting to consider the cumulative impacts of pesticide residues or their prolonged detrimental effects.

Risk is commonly conceptualized as the product of probability and consequence (38), encompassing the likelihood of a hazard causing harm and the severity of that harm (39). In this context, the risk index for pesticide residue in vegetables was developed based on the maximum residue limit (MRL) of pesticides, factoring in both the probability and severity of risk events, aligned with the fundamental principles of dietary risk assessment. This paper methodically measures and evaluates the risk status of pesticide residues in vegetables throughout China, leveraging sampling data from 294,703 vegetable batches across 30 provinces, as published by the Market Supervision Administration between 2021 and 2023. The potential innovations of this study are as follows: First, in contrast to previous studies that rely on small sample sizes, this study consolidates and integrates inspection data from 294,703 samples released by market supervision authorities across 30 provinces (including municipalities and autonomous regions), thereby constructing a comprehensive and standardized database for vegetable safety supervision and sampling, which significantly addresses the limitations in data representativeness found in existing research. Second, diverging from traditional dietary risk assessment and risk measurement methodologies that focus solely on the likelihood or severity of outcomes, this paper introduces and quantifies a pesticide residue risk index that comprehensively accounts for both the probability and severity of risk occurrences for the first time. This enhancement bolsters the scientific validity and credibility of the measurement results, and it advances the prevailing methodologies for assessing and measuring pesticide residue risks. Given the escalating structural mismatch between vegetable supply and demand in China, this research holds substantial practical significance and applicability. To begin with, the findings provide valuable tools and a scientific foundation for governmental regulatory efforts, optimizing the distribution of regulatory resources. In addressing complex regulatory subjects, this study supports the development of scientifically sound food safety supervision risk assessments, inspection plans, and random testing protocols, thus minimizing the arbitrariness of inspections and underpinning the logical arrangement of regulatory priorities. Moreover, by addressing the information asymmetry characteristic of pesticide residues, this study equips consumers with detailed residue data for various vegetables, aiding them in making informed consumption choices based on their personal circumstances. This, in turn, fosters a “bottom-up” mechanism of compliance in vegetable safety production, propelled by consumers “voting with their feet.”

## 2 Data and methods

### 2.1 Data

#### 2.1.1 Data sources

The data utilized in this research are sourced from the food supervision and random inspection records officially disclosed by provincial-level Market Supervision and Administration Bureau across China. According to the *Regulations of the People’s Republic of China on the Disclosure of Government Information (2019 revision)* and the *Measures for the Administration of the Disclosure of Food Safety Information*, regulatory authorities are legally obligated to proactively release food safety inspection results. These data are published via official websites or designated platforms at all administrative levels, following annual or quarterly sampling plans. The resulting datasets are highly authoritative, standardized, and publicly accessible. This study compiled 294,703 vegetable sampling records from 30 provincial-level administrative regions—including municipalities and autonomous regions—collected between 2021 and 2023. These records cover 52 commonly consumed vegetable categories and serve as the foundation for constructing a comprehensive national database reflecting vegetable safety in the circulation stage. The time frame of 2021 to 2023 was selected because China initiated a three-year “Pesticide Control” campaign in 2021 (40).

Compared with existing research, the vegetable supervision and sampling database offers several notable advantages: *First*, the data are authoritative and reliable, having been officially released by local regulatory agencies based on verified testing outcomes. *Second*, the datasets are updated regularly, reflecting dynamic trends in pesticide residue risks through timely publication of inspection results. *Third*, the sampling and inspection process are scientifically rigorous. Vegetable sampling inspection in accordance with the annual *Detailed Rules for the Implementation of National Food Safety Supervision and Sampling Inspection* and the *General Guidelines for Food Sampling Inspection (GB/T 30642–2014)*. Samples are grouped by identical type, origin, vendor, and production or purchase date, ensuring both representativeness and non-duplication. Laboratory testing strictly adheres to national standards, including the *Maximum Residue Limits for Pesticides in Food (GB 2763)*, *Limits of Contaminants in Food (GB 2762),* and additional sector-specific guidelines (the standard system NY/T of the Ministry of Agriculture and Rural Affairs and the inspection and quarantine industry standard system SN/T, etc.). These ensure methodological consistency and scientific integrity throughout the detection process. In short, the scientific nature of vegetable sampling data is ensured in both sampling and inspection.

#### 2.1.2 Data processing

The raw data retrieved from the official websites of provincial Market Supervision and Administration Bureaus encompass structured Excel files, semi-structured Word/PDF documents, and unstructured web-based texts. Key variables include the province of origin, vegetable category, inspection batch, pesticide types tested, detected concentrations, and corresponding maximum residue limits (MRLs) as defined by national standards. To ensure data standardization and analytical usability, a unified protocol was adopted for data extraction and cleaning. The specific data processing flow mainly includes the following three stages.

In the initial stage, all records pertaining to fresh vegetable inspections were screened and classified. Vegetable names were harmonized according to the *Classification and Code of Fresh Vegetables (SB/T 10029–2012)* to ensure cross-regional consistency. In the pesticide residue data processing phase, the pesticide types detected in each sample and their corresponding concentrations were extracted, with extreme outliers removed. Referring to national food safety standards—primarily *GB 2763 (Maximum Residue Limits for Pesticides in Food)* and *GB 2762 (Limits of Contaminants in Food)*—the corresponding MRLs for each pesticide-vegetable combination were retrieved and matched. The measured values were then compared to their respective MRLs to determine compliance. Samples containing banned pesticides or exhibiting residue concentrations above the permissible limits—thereby indicating potential acute health risks (41) —were classified as non-compliant. In the final cleaning phase, entries with missing values or inconsistent pesticide nomenclature were removed, and all concentration units were normalized to mg/kg. The finalized dataset comprises 294,703 samples from 30 provincial-level administrative regions and 52 vegetable categories, including 7,538 batches identified as non-compliant.

Furthermore, data cleaning, indicator construction, and risk assessment in this study were primarily conducted using Excel 2021 and Stata 17.

### 2.2 Methods

#### 2.2.1 Measurement method of pesticide residue risk index

##### 2.2.1.1 Pesticide residue risk index

Risk is defined as the product of the probability of a risky event and its consequences (42, 43). While risk types are varied, the environmental factors, probabilities, and potential outcomes tend to be similar (44). Food safety risk depends on the possibility and severity of adverse health consequences (45). FAO (45) defines risk as a function of the probability and severity of adverse effects that food may have on human health. Building on this, the present study defines pesticide residue risk as the product of its probability of occurrence (P) and severity of harm (S), and accordingly constructs a Pesticide Residue Risk Index (IR), as outlined in Equation 1:


(1) IR=P×S

Further, the pesticide residue risk index of vegetable *j* in Province *i*: $IR_{\mathrm{ij}}=P_{\mathrm{ij}}\times S_{\mathrm{ij}}$ ; The risk of pesticide residues in vegetable *j*: ${\mathrm{IR}}_{j}=P_{j}\times S_{j}$ ; The risk of pesticide residues in vegetables in Province *i*: ${\mathrm{IR}}_{i}=P_{i}\times S_{i}$ . Among them, the specific measurements of *P* and *S* are as follows.

##### 2.2.1.2 The probability of occurrence of pesticide residue risk

The probability of the occurrence of a risk is the quantification of the uncertainty of the occurrence of an event. As the result of producers’ behavior selection under multi-party regulation (9), the unqualified rate can basically reflect the probability of pesticide residue occurrence in vegetables. The probability of pesticide residue exceedance for vegetable category *j* in province *i* is formally defined in Equation 2.


(2) $$P_{\mathrm{ij}}=\frac{M_{\mathrm{ij}}}{N_{\mathrm{ij}}}\times 100\%$$

In the formula, *N_ij_* denotes the total number of sampled batches of vegetable category *j* in province *i*, while *M_ij_* indicates the number of batches of vegetable *j* in Province *i* that excessive pesticide residues.

To comprehensively evaluate the probability of pesticide residue risk across various vegetable categories and provinces, this study employs a direct weighting approach to compute risk probabilities at both the category and regional levels, as defined in Equation 3. Among them, *P_j_* represents the occurrence probability of pesticide residue risk in vegetable *j*; *P_i_* represents the occurrence probability of pesticide residue risks in vegetables of Province *i*.


(3) $$\begin{array}{l} P_{j}=\frac{\sum_{i=1}^{I_{j}}M_{\mathrm{ij}}}{\sum_{i=1}^{I_{j}}N_{\mathrm{ij}}}\\ P_{i}=\frac{\sum_{j=1}^{J_{i}}M_{\mathrm{ij}}}{\sum_{j=1}^{J_{i}}N_{\mathrm{ij}}} \end{array}$$

In the formula, *I_j_* denotes the total number of provinces where vegetable category *j* has been randomly inspected, while *J_i_* denotes the number of vegetable categories sampled and inspected in province *i*.

The *Outline for Building a Powerful Country with Quality* (46) proposes that the pass rate for food sampling inspections should exceed 98%, which implies that the noncompliance rate should generally remain below 2%. In line with this regulatory benchmark, this study adopts 2% as the acceptable upper threshold for the probability of pesticide residue risk occurrence.

##### 2.2.1.3 The severity of harm of pesticide residue risk

To rigorously assess the severity of harm posed by pesticide residues in vegetables, this study adopts the Maximum Residue Limits (MRLs) specified in national food safety standards as the evaluation benchmark and constructs a dimensionless hazard index, S, based on the ratio of the detected concentration to the corresponding MRL (i.e., S = C/MRL). For a given pesticide, the greater the extent to which its residue concentration exceeds the MRL, the higher the associated level of risk. However, for different pesticides, even with identical concentrations, their toxicity profiles differ, making direct comparison of harm infeasible. The standardization of residue concentrations relative to their MRLs allows for meaningful cross-pesticide comparisons. When *S* = 1, it indicates that C equals the MRL, which is the acceptable maximum degree of hazard. *S* > 1, and the larger the value, the higher the degree of potential hazard. Furthermore, the calculation of pesticide residue hazard levels adheres to the “worst-case scenario” principle in risk assessment, thereby minimizing the likelihood of underestimating potential risks. The specific computational steps are outlined as follows:

① First, extract the maximum hazard level (*S_ijn_*) among all over-limit pesticide items in each individual sample, as presented in Equation 4.
② Subsequently, calculate the average value of pesticide residue hazard levels across all samples of vegetable type *j* in province *i*, resulting in the overall pesticide residue hazard level (*S_ij_*), as demonstrated in Equation 5:

(4) $$S_{\mathrm{ijn}}=\max_{k}(\frac{C_{\text{ijnk}}}{\mathrm{MR}L_{\mathrm{jk}}})$$

(5) $$S_{\mathrm{ij}}=\frac{1}{N_{\mathrm{ij}}}\sum_{n=1}^{N_{\mathrm{ij}}}S_{\mathrm{ijn}}$$

In Equation 4, *C_ijnk_* denotes the actual detected concentration of pesticide *k* in the *n*-th randomly sampled batch of vegetable type *j* in province *i*. *MRL_jk_* refers to the maximum permissible residue limit for pesticide *k* in vegetable type *j*, as established by national food safety regulations. The MRL value for a specific pesticide in a given vegetable category is consistent across all batches, meaning that *MRL_ijnk_* equals *MRL_jk_*. In Equation 5, *N_ij_* represents the total number of sampled batches of vegetable category *j* in province *i*.

③ Based on the computed pesticide residue hazard level (*S_ij_*) for vegetable category *j* in province *i*, this study further applies a direct weighting approach to derive the aggregated hazard level of each vegetable type at the national level (*S_j_*) and of all vegetables within each province (*S_i_*). As shown in Equation 6. Among them, *S_j_* represents the degree of pesticide residue hazard of vegetable *j* across the country; *S_i_* represents the overall pesticide residue hazard degree of vegetables in Province *i*.

(6) $$\begin{array}{l} S_{j}=\frac{\sum_{i=1}^{I_{j}}S_{\mathrm{ij}}\times N_{\mathrm{ij}}}{\sum_{i=1}^{I_{j}}N_{\mathrm{ij}}}\\ S_{i}=\frac{\sum_{j=1}^{J_{i}}S_{\mathrm{ij}}\times N_{\mathrm{ij}}}{\sum_{j=1}^{J_{i}}N_{\mathrm{ij}}} \end{array}$$

In the formula, *I_j_* represents the total number of provinces where *j* vegetables were sampled, and *J_i_* represents the number of vegetable types sampled in Province *i*.

By multiplying the acceptable maximum probability of pesticide residue occurrence 2% by the maximum acceptable hazard level 1, the threshold risk index is obtained: IR_0_ = 0.02. This threshold serves as a reference point for classifying risk levels. An IR value below 0.02 indicates a low-risk range for pesticide residues, whereas an IR value equal to or exceeding 0.02 denotes a high-risk level.

#### 2.2.2 Spatial center of gravity measurement of pesticide residue risk

To further examine the spatial dynamics of pesticide residue risks in Chinese vegetables, this study calculates the spatial center of gravity based on provincial-level risk indices. It also analyzes the spatial distribution patterns of overall and specific vegetable categories to trace the diffusion trajectories and directional shifts of pesticide risks over time.

##### 2.2.2.1 Spatial center of gravity

The spatial coordinates (longitude and latitude) of the center of gravity are computed according to Equation 7, where *Long_t_* and *La_t_* represent the longitude and latitude of the center in year *t*, respectively.


(7) $$\begin{array}{l} {\text{Long}}_{t}=\sum_{i=1}^{n}{\text{Long}}_{i}(IR_{\mathrm{it}/\mathrm{ijt}}/\sum_{i=1}^{n}IR_{\mathrm{it}/\mathrm{ijt}})\\ {\mathrm{La}}_{t}=\sum_{i=1}^{n}{\mathrm{La}}_{i}(IR_{\mathrm{it}/\mathrm{ijt}}/\sum_{i=1}^{n}IR_{\mathrm{it}/\mathrm{ijt}}) \end{array}$$

In the formula, *Long_i_* and *La_i_* denote the longitude and latitude coordinates of province *i*, respectively, with the provincial capital city used as the geographic reference point. *IR_it/ijt_* denotes the pesticide residue risk index of either overall vegetables (*IR_it_*) or vegetable category *j* (*IR_ijt_*) in province *i* during year *t*. Specifically, *IR_it_* is used to calculate the spatial center of gravity for overall vegetables, while *IR_ijt_* is used for specific vegetable categories.

##### 2.2.2.2 The shift of the center of gravity in space

Based on the calculated coordinates of the spatial center of gravity, this study further computes the annual transfer distance (*D_t_*) and transfer direction (*A_t_*), where 0° represents due north and angles are measured clockwise. *D_t_* captures the displacement magnitude of the risk centroid, reflecting spatial volatility, while *A_t_* reveals risk diffusion or aggregation paths. The calculation formulas are as follows:


(8) $$\begin{array}{l} D_{t}=\sqrt{{\left({\text{Long}}_{t}-\mathrm{Lon}g_{t-1}\right)}^{2}+{\left({\mathrm{La}}_{t}-La_{t-1}\right)}^{2}}\\ A_{t}=\mathrm{rac}\tan(\frac{{\text{Long}}_{t}-\mathrm{Lon}g_{t-1}}{{\mathrm{La}}_{t}-La_{t-1}}) \end{array}$$

## 3 Results and discussion

### 3.1 Descriptive statistical analysis of the data

To illustrate the overall status of pesticide residue risks in vegetables across China, this study conducts a descriptive statistical analysis of key risk indicators based on province-vegetable category combinations, as shown in Table 1.

**Table 1:** Descriptive statistical analysis.

Variable	Obs	Mean	Std. dev.	Min	Max
P_ij_	775	0.02	0.04	0	0.29
S_ij_	775	3.73	5.74	0	47.67
IR_ij_	775	0.15	0.33	0	4.37

“Variable” represents the name of a variable; “Obs” denotes the number of observed variables; “Mean” denotes the sample mean value; “Std. dev.” denotes the standard deviation of the sample; “Min” denotes the minimum value of the sample; “Max” denotes the maximum value of the sample.

Source: the author obtained it through a simple summary and analysis of the data in the supervision and random inspection database.

The vegetable sampling database includes 294,703 records from 30 provinces (including autonomous regions and municipalities directly under the central government) spanning 2021 to 2023, covering 52 vegetable categories. Among these, 7,538 batches were classified as non-compliant. Following aggregation by province-vegetable category combinations, a total of 775 analytical units were generated, forming the basis for the calculation of the indicators *P_ij_*, *S_ij_*, and *IR_ij_*. Regarding *P_ij_*, the mean is 0.02 and the maximum is 0.29, indicating an overall low probability of exceedance with relatively limited variation. The average *S_ij_* is 3.72, with a maximum value of 47.67, reflecting substantial heterogeneity in hazard levels across provinces and vegetable types. Notably, when *P_ij_* = 0, no pesticide exceedance was detected in vegetable type *j* in province *i*, and S_ij_ is accordingly 0. When *P_ij_* > 0, *S_ij_* typically exceeds 1. As for *IR_ij_*, the mean is 0.15 and the maximum is 4.37, suggesting that the overall pesticide residue risk in Chinese vegetables remains relatively low. However, considerable heterogeneity and potential structural concentration may exist across regional and category dimensions.

### 3.2 Type distribution of pesticide residue risk in vegetables

#### 3.2.1 Sampling of different kinds of vegetables

Analysis of the data from vegetable supervision and random inspections reveals that a total of 52 vegetable types were involved. Among them, the sampling batches of 11 kinds of vegetables such as coriander and okra were less than 10 batches. As illustrated in Table 2, based on the proportion of sampling batches, the top five vegetable varieties from 2021 to 2023 were pepper (34,475 batches), Chinese cabbage (29,299 batches), tomato (28,721 batches), eggplant (26,328 batches), and celery (22,615 batches), collectively accounting for 47.99% of the total vegetable sampling batches. Conversely, the vegetables with the smallest sampling proportions were edible snow peas (35 batches), bottle gourd (38 batches), amaranth (47 batches), colocasia esculenta (49 batches), and mustard (81 batches), each representing less than 1% of the total vegetable sampling batches.

**Table 2:** Sampling situation of kinds of vegetables.

Rank	VT	N_j_	PSB-j
1	Pepper	34,475	11.70%
2	Chinese Cabbage	29,299	9.94%
3	Tomato	28,721	9.75%
4	Eggplant	26,328	8.93%
5	Celery	22,615	7.67%
6	Potato	20,162	6.84%
7	Cowpea	17,199	5.84%
8	Leek	16,526	5.61%
9	Ginger	14,556	4.94%
10	Bean Sprout	12,928	4.39%
11	Cucumber	11,392	3.87%
12	Radish	11,369	3.86%
13	Leaf-Used Lettuce	8,657	2.94%
14	Spinach	8,426	2.86%
15	Mushroom	6,685	2.27%
16	Head Cabbage	6,634	2.25%
17	Yam	4,849	1.65%
18	Kidney Bean	4,755	1.61%
19	Lotus Root	2,239	0.76%
20	Shallot	2023	0.69%
21	Balsam Pear	911	0.31%
22	Onion	719	0.24%
23	Sweet Potato	308	0.10%
24	Broccoli	308	0.10%
25	Garlic Sprout	267	0.09%
26	Lettuce	247	0.08%
27	*Cucurbita Pepo*	239	0.08%
28	Cauliflower	227	0.08%
29	Day-Lily Flower	218	0.07%
30	Lettuce	205	0.07%
31	Pumpkin	179	0.06%
32	Wax Gourd	170	0.06%
33	Water Spinach	160	0.05%
34	Chrysanthemum	138	0.05%
35	Luffa	127	0.04%
36	Garlic	124	0.04%
37	Mustard	81	0.03%
38	*Colocasia Esculenta*	68	0.02%
39	Amaranth	49	0.02%
40	Bottle Gourd	47	0.01%
41	Snow Peas	38	0.01%
42	Others	35	0.02%
	N	294,703	

The “Rank” in the first column is sorted according to the number of batches of vegetables sampled - from highest (1) to lowest (42). “Other” in the form refers to the types of vegetables with no more than 10 batches. Specifically, this category includes 11 types of vegetables: Coriander, Houttuynia, Okra, Chinese toon, Water Chestnut, Green Olive, Fiddlehead Ferns, Pepper Bud, Portulaca oleracea, Sagittaria sagittifolia, and Chicory, totaling 68 batches, which accounts for 0.02% of the total sampled batches.

“VT” denotes Vegetable Type; “N_j_” denotes the number of random inspection batches of vegetable j; “PSB-j” denotes the proportion of the sampled inspection batch of vegetable *j* to all sampled inspection batches, that is, N_j_/N; “N” denotes the random inspection batches of all vegetables.

Source: the author calculated and summarized the data from the supervision and random inspection of vegetables.

#### 3.2.2 Pesticide residue risk of different types of vegetables

From the measurement results of pesticide residue risk of different kinds of vegetables, there are significant differences in pesticide residue risk of different kinds of vegetables. As shown in Table 3, the risk index of pesticide residues for 21 vegetable types exceeded 0.02, placing them in the high-risk category. Notably, leek, cowpea, ginger, celery, and onion exhibited the highest levels of pesticide residues, accounting for 20.37, 16.85, 15.63, 10.94, and 1.67% of the total batches with excessive pesticide residues, respectively. The risk index of pesticide residues in eight vegetables, such as wax gourd, Chinese cabbage, and potato, was below 0.02, indicating a low-risk range. The proportion of excessive batches in these vegetables was 1.23% of the total excessive batches. The interval between the last pesticide application and the harvest in vegetable cultivation must exceed the pre-harvest interval (PHI) (PHI is the interval of time that must be waited between the last application of a pesticide and the harvest of a crop). However, in actual agricultural practices, unforeseen pest outbreaks and the unpredictable timing of vegetable ripening often hinder farmers’ adherence to these safety intervals (47). Deviations from these prescribed intervals are closely linked to the eventual levels of pesticide residues found in agricultural products (48, 49). Additionally, inherent growth characteristics of vegetables, such as differences in growth cycles, morphological structures, and pesticide absorption capacities, lead to significant variations in pesticide residue levels among different types of vegetables (50).

**Table 3:** Risk of pesticide residues in subdivided types of vegetables.

Rank	VT	P_j_	S_j_	IR_j_
High-Risk				
1	Leek	9.29%	8.779	0.815
2	Cowpea	7.38%	8.265	0.610
3	Ginger	8.09%	4.618	0.374
4	Celery	3.64%	8.222	0.300
5	Shallot	6.23%	3.777	0.235
6	Spinach	2.49%	7.659	0.191
7	Bean Sprout	0.82%	17.592	0.144
8	Kidney Bean	2.23%	6.123	0.136
9	Pepper	2.91%	3.655	0.106
10	Chinese Cabbage	1.35%	7.625	0.103
11	Leaf-Used Lettuce	1.93%	5.064	0.098
12	Yam	1.59%	5.309	0.084
13	Sweet Potato	1.95%	3.837	0.075
14	Luffa	2.36%	3.072	0.073
15	Lettuce	1.46%	3.581	0.052
16	Balsam Pear	0.77%	5.067	0.039
17	Eggplant	1.11%	3.179	0.035
18	Radish	0.43%	7.57	0.033
19	Chrysanthemum	1.45%	1.825	0.026
20	Cucumber	0.50%	4.764	0.024
21	Mushroom	0.33%	6.871	0.023
Low-Risk				
22	Wax Gourd	0.59%	2.55	0.015
23	Head Cabbage	0.26%	4.44	0.011
24	Potato	0.16%	4.374	0.007
25	*Cucurbita Pepo*	0.42%	1.54	0.006
26	Lettuce	0.40%	1.45	0.006
27	Broccoli	0.32%	1.8	0.006
28	Tomato	0.13%	3.011	0.004
29	Lotus Root	0.09%	1.925	0.002

Considering that the fact that the number of sampling batches was too small might not reflect the real situation of pesticide residues in this vegetable, 36 vegetable varieties with the total sampling batches exceeding 100 in 3 years were screened in this paper, among which seven vegetables including onion, garlic stalk, cauliflower, day lily, pumpkin, water spinach and garlic were all found to have pesticide residues exceeding the standard, with a high safety level, while the remaining 29 vegetables were all found to have pesticide residues exceeding the standard.

The “Rank” in the first column is based on the vegetable pesticide residue risk index—from highest (1) to lowest (29).

“VT” denotes Vegetable Type; “*P_j_*” denotes the probability of pesticide residue risk occurrence in vegetable *j*; “*S_j_*” denotes the severity of the pesticide residue risk of vegetable *j*; “IR_j_” denotes the pesticide residue risk of vegetable *j*.

Source: the authors calculated the results using the vegetable supervision and inspection data, following the formulas outlined in Section 2.2.

**Figure 1:**
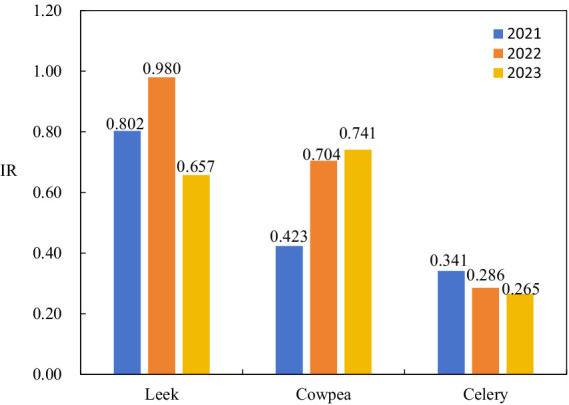
Pesticide residue risk index of leek, cowpea and celery.

Since 2021, a national initiative has been launched to address pesticide residues in edible agricultural products, with a specific focus on mitigating residues in celery, leeks, and cowpeas. Despite some progress, the effectiveness of these measures has varied, as evidenced in Figure 1. The risk associated with pesticide residues in celery has marginally declined, whereas for leeks, it has shown variability. Conversely, the risk for cowpeas has escalated, underscoring an increasing safety concern. Leeks and cowpeas, classified as “multi-harvest” vegetables, are characterized by their capacity for multiple harvests within a single growth cycle and relatively brief harvesting periods. Consequently, such production traits substantially heighten the likelihood of overlap between pesticide safety intervals and successive harvesting cycles. This overlap is particularly pronounced when farmers are driven by economic incentives to maximize short-term yields, thereby intensifying the risk of pesticide residues. Research has also indicated variability in the residual presence of pesticides within different parts of the same vegetable, typically diminishing from leaves to roots (51–53), which results in an elevated risk of excessive pesticide residues in leafy vegetables such as celery and leeks. Additionally, while certain vegetables exhibit a low probability of pesticide residue presence, the severe potential harm escalates their risk index. For instance, bean sprouts have a pesticide residue probability of merely 0.82%, yet their risk and harm degree are the highest among all vegetables at 17.592, resulting in a risk index of 0.144, which is 7.20 times the acceptable level. The pesticide residue status of such vegetables warrants considerable attention.

### 3.3 Regional distribution of pesticide residue risk in vegetables

#### 3.3.1 Sampling status of vegetables in different regions

From the perspective of regional sampling inspection, as shown in Table 4, the top 5 provinces with a large proportion of vegetable sampling inspection batches in the country are Chongqing (39,327 batches), Guizhou (20,410 batches), Yunnan (20,288 batches), Beijing (19,835 batches) and Shandong (19,823 batches) in descending order. It accounts for 40.61% of the total vegetable sampling batches, of which Chongqing, which has the most sampling batches, has the largest per capita vegetable consumption, with an average annual per capita vegetable consumption of 147.03 kg from 2021 to 2023. However, judging from the intensity of vegetable sampling per capita in the region (sampling batches per 10,000 people), the top five provinces with the largest per capita sampling batches are Chongqing (12.27), Beijing (9.06), Qinghai (7.47), Hainan (6.09) and Ningxia (6). From the perspective of the unqualified situation of regional vegetable sampling, the same as the previous research on the excessive pesticide residues of fruits and vegetables in various provinces, the vegetable supervision sampling data collected from 2021 to 2023 in this study also found that Shandong, Chongqing and Henan had the largest number of batches of pesticide residues exceeding the standard (20), 835, 687 and 513 batches, respectively. However, contrary to the explanation, there are more batches of vegetables in the above three regions, so there are more unqualified batches, and not just because of the larger planting area of vegetables in the regions. And in the market environment of large circulation of vegetables across the country, vegetables grown in the region are not only sold locally.

**Table 4:** Sampling of vegetables in different regions.

Rank	Province	N_i_	PSB-i	NBP	M_i_
1	Chongqing	39,327	13.34%	12.27	687
2	Guizhou	20,410	6.93%	5.29	305
3	Yunnan	20,288	6.88%	4.3	685
4	Beijing	19,835	6.73%	9.06	453
5	Shandong	19,823	6.73%	1.95	835
6	Henan	18,298	6.21%	1.84	513
7	Xinjiang	12,094	4.10%	4.68	271
8	Shaanxi	11,129	3.78%	2.82	108
9	Guangxi	10,501	3.56%	2.1	172
10	Guangdong	9,786	3.32%	0.78	210
11	Anhui	9,231	3.13%	1.51	181
12	Heilongjiang	8,422	2.86%	2.64	150
13	Hunan	7,633	2.59%	1.15	268
14	Sichuan	7,208	2.45%	0.86	196
15	Jiangsu	6,824	2.32%	0.81	313
N			294,703		
16	Shanxi	6,818	2.31%	1.95	335
17	Fujian	6,713	2.28%	1.62	307
18	JiLin	6,381	2.17%	2.65	186
19	Hainan	6,138	2.08%	6.09	228
20	Zhejiang	5,861	1.99%	0.91	252
21	Hubei	5,608	1.90%	0.97	171
22	Jiangxi	5,045	1.71%	1.12	150
23	Liaoning	4,464	1.51%	1.05	68
24	Qinghai	4,422	1.50%	7.47	146
25	Ningxia	4,318	1.47%	6	46
26	Hebei	4,256	1.44%	0.57	17
27	Tianjin	4,229	1.44%	3.05	79
28	Inner Mongolia	3,628	1.23%	1.51	102
29	Shanghai	3,020	1.02%	1.21	29
30	Gansu	2,993	1.02%	1.2	75
M			7,538		

The “Rank” in the first column is based on the sampling of vegetables in each province - from highest (1) to lowest (30).

“N_i_” denotes the batch of vegetables sampled and inspected in Province *i*; “PSB-*i*” denotes the proportion of vegetable sampling inspection batches in Province *i* to all sampling inspection batches, that is, N_i_/N; “NBP” denotes Number of inspection batches per 10,000 people in Province *i*; “M_i_” denotes the unqualified batches of vegetables in Province *i*; “N” denotes the random inspection batches of all vegetables; “M” represents all the unqualified batches of vegetables.

Source: With the exception of the population data for each province used in the calculation of ‘NBP, ‘which is sourced from the ‘China Statistical Yearbook,’ all other data were calculated and summarized by the authors using vegetable supervision and sampling inspection data.

#### 3.3.2 Pesticide residue risk of vegetables in different regions

The level of economic development in different regions of China is obviously different, and the situation of pesticide residues in vegetables is also different (54). We comprehensively considered the probability and harm degree of regional vegetable pesticide residue risk, and measured the risk of vegetable pesticide residue in various regions of China, and the results were shown in Table 5. Except for Tianjin, the risk indices for pesticide residues in vegetables from other provinces were above 0.02, indicating a high-risk range. The top five provinces in terms of risk index were Jiangsu, Jilin, Hubei, Hainan, and Heilongjiang, respectively. The proportions of random vegetable batches from these regions were 2.32, 2.17, 1.90, 2.08, and 2.86%, respectively. Ningxia, Yunnan, Shaanxi, Gansu, and Tianjin ranked among the bottom five provinces in terms of risk index, with vegetable sampling batches accounting for 3.56, 1.47, 6.88, 3.78, and 1.02% of the total sampling batches, respectively.

**Table 5:** Pesticide residue risk status of vegetables in each province.

Rank	Province (*i*)	IR_i_
1	Jiangsu	0.380
2	Jilin	0.329
3	Hubei	0.297
4	Hainan	0.266
5	Heilongjiang	0.256
6	Henan	0.247
7	Fujian	0.229
8	Shanxi	0.226
9	Qinghai	0.225
10	Shandong	0.224
11	Hunan	0.219
12	Zhejiang	0.203
13	Inner Mongolia	0.193
14	Guizhou	0.190
15	Beijing	0.190
16	Shanghai	0.171
17	Jiangxi	0.150
18	Sichuan	0.133
19	Liaoning	0.130
20	Chongqing	0.114
21	Xinjiang	0.110
22	Guangdong	0.106
23	Anhui	0.099
24	Hebei	0.089
25	Guangxi	0.085
26	Ningxia	0.080
27	Yunnan	0.077
28	Shaanxi	0.073
29	Gansu	0.048
30	Tianjin	0.018

The “Rank” in the first column is based on the vegetable pesticide residue risk index of the province - from highest (1) to lowest (30).

“IR_i_” denotes the pesticide residue risk index of vegetables in Province *i*.

Source: the authors calculated the results using the vegetable supervision and inspection data, following the formulas outlined in Section 2.2.

As a pivotal element in ensuring the quality and safety of fruits and vegetables, government regulation is intimately connected to the risk of pesticide residues (55–57). Economically more developed provinces usually have more regulatory resources and can carry out more frequent pesticide residue testing and stricter control, but due to the larger market size and wide circulation scope, the regulatory pressure is relatively high. Research shows that for every 1% increase in government sampling, the rate of excessive pesticide residues can decrease by 0.28% (9). By comparing the “vegetable sampling batches per 10,000 people” across different provinces, it is evident that Jiangsu and Hubei, which have high pesticide residue risks, have sampling batches per 10,000 people that are all below 1. In contrast, the market size of low-risk provinces such as Ningxia and Shaanxi is small, and the relatively concentrated regulatory resources may help effectively control risks. This difference in the allocation of regulatory resources leads to different levels of pesticide residue control in vegetables.

**Figure 2:**
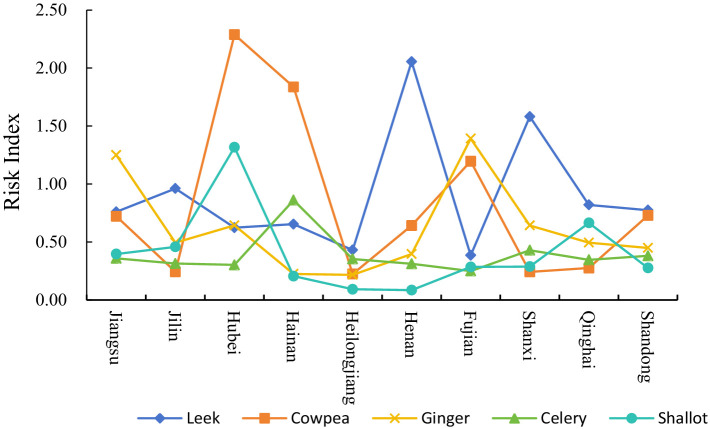
Pesticide residue risk of specific vegetable types in different regions.

**Figure 3:**
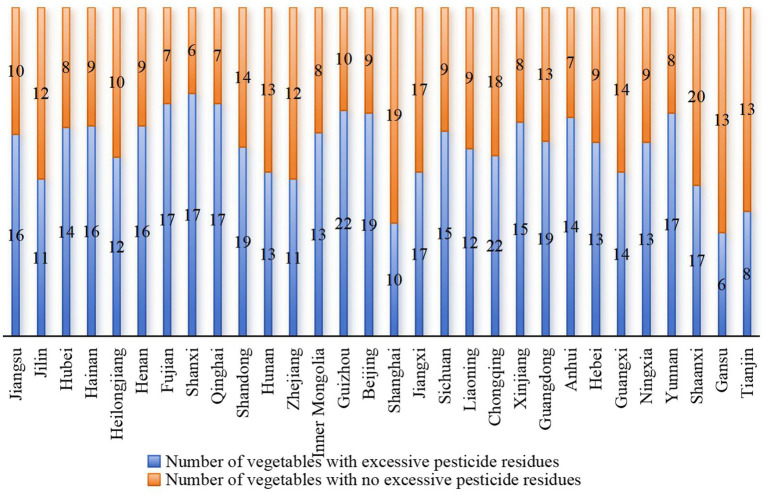
Types of vegetables sampled by provinces in China and types of vegetables with excessive pesticide residues.

The risk of pesticide residues in vegetables in different provinces also showed distinct characteristics. The risk of pesticide residues of 5 kinds of vegetables, including leek, cowpea, ginger, celery and shallot, was generally higher in all provinces. The pesticide residue risk status of 5 vegetables in 7 regions (from high to low) is shown in Figure 2. The risk of pesticide residues in leek and cowpea varied greatly among different regions, and the risk of pesticide residues in leek and cowpea in Henan and Shanxi was the highest, while the risk of pesticide residues in cowpea in Hubei, Hainan and Fujian was higher. For celery, the risk of pesticide residues is roughly the same in different regions. Shallot had a higher risk of pesticide residue in Hubei, but there was not much difference in risk in other regions. As shown in Figure 3 below, in addition to the top 5 vegetables with higher risk, other high-risk vegetable types in each province also showed significant regional specificity. Specifically, 16 of the 26 kinds of vegetables sampled in Jiangsu were detected with excessive pesticide residues, among which yam and shallot had the highest risk of pesticide residues (except leek, cowpea, ginger and celery), the risk index was 0.478 and 0.396 respectively, and the probability of exceeding the standard exceeded 10%. 31 batches of yam were sampled. 7 batches of samples were detected with excessive pesticide residues; Among the 23 kinds of vegetables sampled in Jilin, 11 kinds of pesticide residues exceeded the standard, among which the risk of pesticide residues was higher in shallot and leaf-used lettuce, the risk index was 0.458 and 0.418, and the multiple of residue exceeded the standard was 11.0 and 26.3, respectively. In Hubei, 14 of 22 kinds of vegetables were found to have excessive pesticide residues, and the risk index of bean sprouts and onion was 1.452 and 1.317, respectively. For different regions, the risk of pesticide residues of various types of vegetables is obviously different, so it is necessary to strengthen the supervision and sampling intensity according to the actual situation.

In addition, to further explore the regional characteristics of pesticide residues in vegetables, this study utilizes province-level risk indices to track changes in the spatial center of gravity of such risks. Based on Equations 7, 8, we compute both the transfer direction (*A_t_*) and transfer distance (*D_t_*) to map the dynamic diffusion trajectories of pesticide residue risks—both overall and by specific vegetable categories—during the period from 2021 to 2023 (see Section 2.2.2 for methodological details). As shown in Table 6, the spatial center of gravity for vegetable pesticide residue risk shifted from (114.48°E, 33.70°N) in 2021 to (113.28°E, 32.97°N) in 2023, clearly indicating a spatial migration of risk from the northeast toward the southwest. This directional shift suggests that pesticide residue risks are gradually becoming more concentrated in southwestern provinces. An analysis of the displacement direction and distance of the top five high-risk vegetable categories reveals that this spatial shift was primarily driven by celery (*D_t_* = 230.25 km), followed by cowpea (*D_t_* = 100.91 km). This trend can be discerned through the transfer direction and distance of the top five high-risk vegetables. The primary driver of this spatial shift in pesticide residue risk was celery, covering a distance of 230.25 km, followed by cowpea, at 100.91 km. Regarding vegetable types, among the top five vegetables with higher pesticide residue risks, ginger stands out with its longer growth cycle, typically lasting between 6 to 12 months. Its low perishability facilitates both storage and transportation. As a significant seasoning and medicinal plant, ginger enjoys widespread market demand and is often transported and sold across regions (58). Consequently, its production and consumption span a broad geographic area, leading to a wider spread of its pesticide residue risk. In contrast, cowpeas and spinach have considerably shorter growth cycles, particularly spinach, which is usually harvested within a few weeks. Due to the high perishability of these vegetables and the strict consumer demand for freshness, they are predominantly distributed within local markets. This localized supply chain effectively reduces circulation distances, limits the spatial spread of pesticide residue risk, and results in a more concentrated risk center.

**Table 6:** Dynamic transfer trajectory of spatial center of gravity of vegetable pesticide residue risk.

VT	2021		2023		Transfer locus	
	Longitude (E)	Latitude (N)	Longitude (E)	Latitude (N)	*A_t_*	*D_t_*
WV	114.48	33.70	113.28	32.97	Northeast to Southwest	137.61
Leek	113.58	34.08	114.80	35.10	Southwest to Northeast	159.65
Cowpea	114.48	29.94	113.60	29.46	Northeast to Southwest	100.91
Ginger	111.88	30.61	113.81	33.57	Southwest to Northeast	375.68
Celery	111.82	33.50	111.44	31.45	Northeast to Southwest	230.25
Spinach	109.41	31.54	109.58	30.45	Northwest to Southeast	122.36

The top five vegetables with the highest pesticide residue risk are leek, cowpea, ginger, celery, and green onion. Since the sampling data for green onion in 2021 and 2022 covers only 4 and 9 provinces respectively, there is a regional selection bias. Therefore, the risk diffusion trajectory of pesticide residue in green onion has not been calculated. To ensure data representativeness, spinach, ranked sixth, is selected in this table to calculate the dynamic diffusion trajectory of the spatial center of gravity for its pesticide residue risk instead. The diffusion distance is measured in kilometers.

“VT” denotes Vegetable Type; “WV” denotes the whole vegetable; “*A_t_*” denotes transfer direction; “*D_t_*” denotes transfer direction.

Source: the author utilized the data in the supervision and random inspection database, combined with the longitude and latitude data of provincial capital cities from the National Basic Geographic Information Center, and calculated it according to the formula in “2.2.”

### 3.4 Esidual risk status of different kinds of pesticides

Although the drug needs of different vegetables are different, from the test results, the types of pesticides exceeding the standard show relatively concentrated characteristics. The main pesticides used in agricultural cultivation are insecticides, fungicides, herbicides and plant growth regulators. According to the overall sampling of vegetables in China from 2021 to 2023, a total of 50 kinds of excessive pesticides were detected in the 52 kinds of vegetables sampled, and the ratio of crop types to pesticide types was always close to 1:1, which was the same as the research results of Zhou, et al. (20). China is considered to be one of the largest pesticide consumers in the world (59), and pesticides and fungicides in vegetables are the most important categories of pesticides exceeding the standard, accounting for more than 90% of the samples exceeding the standard.

**Figure 4:**
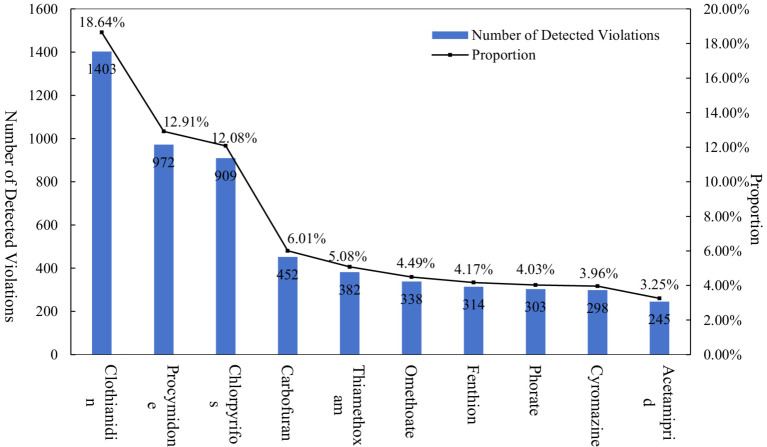
Number of detected pesticide violations and their proportions (TOP 10).

As shown in Figure 4, the most detected pesticides in Brazil are the insecticides imidacloprid and the fungicides pentazolol and carbendazim (29). In Chinese vegetables, the number of samples of clothianidin, procymidone and chlorpyrifos exceeded the standard was the highest, with 1,403, 972 and 909 batches, respectively, accounting for 43.63% of the total samples with exceeded pesticide residues. Specifically: ① Clothianidin was detected exceeding the limit in seven vegetables, with ginger and pepper accounting for 72.7% of the samples exceeding the limit. The unique absorption mechanisms rendered ginger and pepper the most affected by clothianidin. Specifically, ginger, being an underground tuber, has a high probability of absorbing residual clothianidin from the soil during its growth; conversely, pepper, with its larger fruit surface area and uneven texture, tends to retain more pesticides. ② Procymidone is a kind of fungicide, and its exceedant quantity accounts for 12.91% of the total exceedant quantity, which is mainly detected in leek. The reasons for excessive procymidone in leek can be summarized as follows: Firstly, the limited choice of pesticides. In China, for a long time, there is only one registered active ingredient of phytomyces cinerea that can be used to prevent gray mold of Chinese leek (60), and long-term use of one pesticide is likely to increase the resistance of diseases and pests (61), which further aggravates the problem of excessive phytomyces in Chinese leek. Secondly, procymidone’s systemic properties enable it to persist in the soil for extended periods. Coupled with the lack of soil improvement measures or extended fallow periods by farmers, this increases the risk of procymidone residue accumulation in leek cultivation. Thirdly, the pre-harvest interval (PHI) for procymidone is relatively long (62), while the growth cycle of leeks is short (63). The PHI for procymidone on leeks is up to 30 days, with national regulations stipulating a maximum of 1–2 applications per season. Leeks are a typical ‘multiple-harvest’ vegetable, with a single harvest cycle usually lasting 20–30 days, which is shorter than the PHI. To ensure timely harvesting, farmers may not strictly adhere to the recommended pesticide application guidelines during actual usage (3). Chlorpyrifos has been banned by the Ministry of Agriculture for vegetable cultivation since 2017. However, sampling results indicate its continued presence in 18 vegetables, including celery, leek, and spinach, highlighting a severe issue with illegal pesticide use. Relevant studies have found that chlorpyrifos exposure is positively associated with lung cancer incidence (64), while insecticides such as clothianidin have been associated with a high risk of liver cancer (65). There is an urgent need to enhance pesticide regulation and management, and to improve farmers’ awareness of standardized pesticide use.

Additionally, the number of pesticide residue types exceeding the standard varied significantly among different vegetables. Cowpea and celery were notable for having more than 20 types of pesticide residues exceeding the standard. Vegetables with 10–20 types of pesticide residues exceeding the standard included pepper (19 types), Chinese cabbage (19 types), leek (17 types), kidney bean (15 types), leaf-used lettuce (14 types), cucumber (14 types), and eggplant (10 types). In contrast, vegetables such as ginger, spinach, and radish had fewer than 10 types of pesticide residues exceeding the standard.

## 4 Conclusions and recommendations

With the modernization of agricultural production and the widespread use of chemical pesticides, pesticide residue risk has become an important public health issue of global concern. Under the effect of “broken window effect” (1, 66), pesticide residue risk is superimposed in conduction. Therefore, scientific assessment of pesticide residue risk and formulation of effective regulatory policies are the key to ensuring food safety. This study comprehensively considers the probability of risk occurrence and the degree of harm, constructs and quantifies the pesticide residue risk, and measures and evaluates the pesticide residue risk of 52 subdivided types of vegetables by using the supervised sampling data of 290,000 vegetables in 30 provinces from 2021 to 2023. The results are as follows:

(1) A total of 52 kinds of vegetables were sampled, among which the top 5 kinds of vegetables with more samples were chili, cabbage, tomato, eggplant and celery, accounting for 47.99% of the total samples of vegetables. Considerable variations were observed in the pesticide residue risk indices across different vegetable varieties. Among these, onions and seven other vegetable varieties were found to be within acceptable limits, exhibiting relatively low pesticide residue risks. Conversely, samples from 29 vegetable varieties, including leeks, were found to exceed regulatory limits, indicating a heightened risk of pesticide residues. Notably, leeks, cowpeas, and celery—key vegetables subject to state regulation—continue to present the highest risks of pesticide residues.
(2) The top 5 regions accounted for 40.61% of the total samples, and the pesticide residue risk of vegetables showed significant heterogeneity at the regional level. The provinces with the highest risk indices include Jiangsu, Jilin, Hubei, Hainan, and Heilongjiang. Conversely, the provinces with the lowest risk indices are Tianjin, Gansu, Shaanxi, Yunnan, and Ningxia. Vegetables such as leeks, cowpeas, ginger, and celery consistently exhibit high-risk characteristics across various provinces. However, other high-risk vegetable varieties did not display a consistent regional pattern. The risk of pesticide residues in vegetables is progressively spreading from the northeast to the southwest.
(3) The categories of pesticide residues exceeding the standard exhibited a pattern of “diversity and relative concentration.” The sampling data revealed that 50 pesticide components exceeded the limit, with residues of clothianidin, procymidone, and chlorpyrifos being the most prominent, comprising 43.63% of all exceedances. Additionally, multiple pesticide components exceeding the standard were frequently detected within the same vegetable, and the types of pesticide residues varied significantly among different vegetables.

In light of the aforementioned conclusions, this paper offers the following policy recommendations:

(1) Enhance the risk assessment framework by implementing a classification management system. Grounded in scientific risk evaluations, bolster dynamic monitoring of pesticide residues in vegetables and periodically update the risk levels associated with various vegetable types. Concurrently, each region should align its classification and management practices with the specific risk profiles of local vegetables, developing tailored risk control standards and inspection frequencies to ensure that high-risk vegetables receive more rigorous oversight.
(2) Develop an information exchange platform and establish a market mechanism. By creating an online query platform for agricultural product compliance certification, consumers can access and verify real-time information on detection and safety levels, thereby mitigating information asymmetry and enabling informed purchasing decisions. Simultaneously, the consumer “vote with the feet” effect will drive a survival-of-the-fittest market mechanism, compelling farmers to enhance vegetable safety standards and fostering industry-wide standardization.
(3) Establish a key pesticide monitoring list and intensify the research and development of environmentally friendly pesticides. Strengthen oversight on high-incidence pesticide components such as clothianidin and procymidone. Concurrently, bolster the development of rapidly degradable, eco-friendly pesticides, and support their market adoption through policy subsidies and tax incentives, gradually replacing conventional pesticides and mitigating risks associated with safety intervals.

## Data Availability

The data used in this study was collected by the research team from government-published food safety supervision and sampling records, which are not stored in an open-access repository. However, the data can be made available by the authors upon reasonable request.
